# Associação entre escolaridade e taxa de mortalidade por dengue no Brasil 

**DOI:** 10.1590/0102-311XPT215122

**Published:** 2023-09-25

**Authors:** Lucas Melo Guimarães, Geraldo Marcelo da Cunha, Iuri da Costa Leite, Ronaldo Ismerio Moreira, Eduilson Lívio Neves da Costa Carneiro

**Affiliations:** 1 Fundação Municipal de Saúde, Teresina, Brasil.; 2 Escola Nacional de Saúde Pública Sergio Arouca, Fundação Oswaldo Cruz, Rio de Janeiro, Brasil.; 3 Instituto Nacional de Infectologia Evandro Chagas, Fundação Oswaldo Cruz, Rio de Janeiro, Brasil.; 4 Instituto Federal de Educação, Ciência e Tecnologia do Piauí, Teresina, Brasil.

**Keywords:** Dengue, Escolaridade, Mortalidade, Dengue, Educational Status, Mortality, Dengue, Escolaridad, Mortalidad

## Abstract

A dengue pode estar associada a variáveis de nível individual, como escolaridade, aumentando o risco de adoecimento. O objetivo deste trabalho é analisar as disparidades da mortalidade por dengue entre os menos e mais escolarizados no Brasil entre os anos de 2010 e 2018. Este é um estudo do tipo ecológico retrospectivo das diferenças na taxa de mortalidade por dengue entre menos e mais escolarizados no Brasil, através das taxas de mortalidade por dengue geral, por idade, sexo e Unidade Federativa (UF). Um procedimento de *bootstrap* e imputação múltipla para a variável escolaridade foram implementados de modo a considerar a estrutura multinível em cada UF dos dados ao longo dos anos. Para cada banco agregado gerado, foi ajustado um modelo de Poisson multinível. A melhoria na escolaridade da população brasileira não refletiu na diminuição da mortalidade por dengue. Houve um aumento na taxa de mortalidade por dengue no Brasil e um crescimento da diferença de taxas de mortalidade entre menos e mais escolarizados. Independentemente do processo de imputação, os resultados mostraram maiores taxas de mortalidade por dengue entre os menos escolarizados. A baixa escolaridade afetou de forma mais pronunciada os mais jovens.

## Abstract (en)

Dengue may be associated with individual level variables, such as schooling, increasing the risk of illness. The objective of this study is to analyze the disparities in dengue mortality among the least and the most educated in Brazil, from 2010 to 2018. This is a retrospective ecological study of the differences in the mortality rate due to dengue between the less and the more educated people in Brazil, according to the mortality rates due to general dengue, by age, sex, and Federative Unit (UF). A bootstrap and multiple imputation procedure for the variable schooling was implemented to consider the multilevel structure of the data from each UF over the years. For each aggregate bank generated, a multilevel Poisson model was adjusted. The improvement in the education level of the Brazilian population did not reflect on the decrease in mortality from dengue. There was an increase in the mortality rate from dengue in Brazil and an increase in the difference in mortality rates between less and more educated. Regardless of the imputation process, the results showed higher mortality rates from dengue among the less educated. Low schooling affected younger people more pronouncedly.

## Abstract (es)

El dengue puede estar asociado a variables de nivel individual como la educación, aumentando el riesgo de enfermarse. El objetivo de este trabajo es analizar las disparidades de la mortalidad por dengue entre las personas con menor y mayor nivel educativo en Brasil entre los años 2010 y 2018. Estudio del tipo ecológico retrospectivo de las diferencias en la tasa de mortalidad por dengue entre las personas con menor y mayor nivel educativo en Brasil, a través de las tasas de mortalidad por dengue general, por edad, género y Unidad Federativa (UF). Se implementó un procedimiento de *bootstrap* y de imputación múltiple para la variable de educación para considerar la estructura multinivel en cada UF de los datos a lo largo de los años. Para cada banco agregado generado, se ajustó un modelo de Poisson multinivel. La mejora en la educación de la población brasileña no se reflejó en la disminución de la mortalidad por dengue. Hubo un aumento en la tasa de mortalidad por dengue en Brasil y un crecimiento de la diferencia en las tasas de mortalidad entre las personas con menor y mayor nivel educativo. Independientemente del proceso de imputación, los resultados mostraron mayores tasas de mortalidad por dengue entre los menos educados. El bajo nivel de educación afectó de forma más pronunciada a los más jóvenes.

## Introdução

A dengue é a doença viral transmitida por mosquitos mais importante do mundo e metade da população está exposta ao risco de desenvolvê-la ^1^^,^^2^. A infecção pode levar a um amplo espectro de sintomas, desde quadros assintomáticos a cenários graves, com necessidade de atendimento médico e hospitalização ^3^^,^^4^. A assistência de saúde prestada e a precocidade com que se inicia o tratamento estão relacionadas ao risco de morte ^5^. No mundo, estima-se uma taxa de mortalidade de 2,5 por 1 milhão de pessoas por ano e houve tendência de queda de 28% entre 2010 e 2016 ^1^. Porém, no Brasil, houve aumento de 500% na taxa de mortalidade entre 2000 (0,4 por 1 milhão de pessoas) e 2015 (2,4 por 1 milhão de pessoas) ^6^.

O adoecimento e a morte por dengue não atingem a população de forma homogênea. Estudos apontam maiores índices de morbimortalidade da doença em populações residentes de regiões com piores condições socioeconômicas ^7^^,^^8^^,^^9^^,^^10^. A possível relação causal de variáveis dos níveis individual e comunitário, como baixa escolaridade, habitação precária, inefetividade política de controle do vetor e condições precárias de saneamento, combinadas com as condições ambientais e climáticas, favorecem a cadeia de transmissão, aumentando o risco de adoecimento das populações mais vulneráveis ^11^^,^^12^^,^^13^.

A educação, de forma geral, promove diversos benefícios para as condições de saúde e longevidade da população ^14^^,^^15^. No Brasil, diversos estudos apontam para uma relação indireta entre mortalidade geral e nível de escolaridade - quanto maior o nível de escolaridade, menor a probabilidade de morte na idade adulta por qualquer motivo ^16^^,^^17^^,^^18^.

Apesar de as evidências apontarem para uma possível relação causal entre baixa escolaridade e incidência de dengue, há uma carência de estudos sobre a associação entre escolaridade e mortalidade por dengue no Brasil e em nível nacional. Isso se deve, provavelmente, aos desafios metodológicos relativos à falta de preenchimento da variável escolaridade no banco de dados sobre mortalidade. Dessa forma, o objetivo deste trabalho é analisar os diferenciais de mortalidade por dengue segundo o nível de escolaridade no Brasil entre 2010 e 2018.

## Métodos

### Tipo e área de estudo

Este é um estudo ecológico retrospectivo que busca compreender as diferenças na mortalidade por dengue entre menos e mais escolarizados no Brasil, de 2010 a 2018 (último ano com informação de mortalidade disponível no momento da pesquisa), mediante as taxas de mortalidade geral por dengue, por idade, sexo e Unidades Federativas (UF). A pesquisa abrange o Brasil, maior país da América do Sul e sexto mais populoso do mundo. Sua população é predominantemente urbana, com maior concentração nas regiões Nordeste e Sudeste. O país é dividido em cinco macrorregiões e 27 UF.

### Numerador

Para calcular a taxa de mortalidade, os dados sobre óbitos foram coletados no Sistema de Informação sobre Mortalidade (SIM) do Ministério da Saúde. As informações das mortes por dengue foram selecionadas considerando a causa básica da Declaração de Óbito (DO), segundo as terminologias da Classificação Internacional de Doenças, 10ª revisão (CID-10), A90 (dengue clássico) e A91 (febre hemorrágica devida ao vírus do dengue), entre 2010 e 2018, para todas as UF do Brasil.

### Denominador

Os dados das populações estratificadas por sexo, idade, escolaridade e UF para cada ano do período de 2010 a 2018 foram inicialmente calculados utilizando-se as informações dos microdados do *Censo Demográfico* de 2010 ^19^ e da *Pesquisa Nacional por Amostra de Domicílios* (PNAD) para 2012-2018 do Instituto Brasileiro de Geografia e Estatística (IBGE) ^20^. Devido a oscilações de natureza aleatória nas estimativas das populações, modelos lineares foram utilizados para o ajuste das populações ao longo dos anos em cada um dos estratos compostos por UF, sexo, idade e escolaridade. Os valores preditos dos modelos foram assumidos como os verdadeiros tamanhos das populações em cada estrato. Devido ao intervalo de tempo relativamente curto deste estudo, essas estimativas mostraram-se bastante plausíveis.

### Modelo estatístico

O não preenchimento geral da variável escolaridade no período de estudo nas DO no Brasil foi de 22,1%. Um procedimento de imputação para essa e demais variáveis foi implementado de modo a considerar a estrutura multinível dos dados, ou seja, óbitos por dengue observados em cada UF ao longo dos anos. Inicialmente, toma-se um conjunto de amostras *bootstrap* do grupo de dados original (incluindo valores ausentes), considerando a estrutura multinível. *Bootstrap* é um método de reamostragem de dados com reposição que permite estimar a variabilidade e, por conseguinte, intervalos de 95% de confiança (IC95%) de estatísticas complexas, com base na distribuição empírica das reamostras ^21^. Posteriormente, os valores faltantes em cada amostra *bootstrap* foram preenchidos por meio de imputação múltipla ^22^, procedimento que envolve a substituição de cada valor ausente por uma série de valores plausíveis, gerando, assim, vários conjuntos de dados completos. Analisados separadamente, esses conjuntos produziram estimativas, as quais foram então combinadas em uma estimativa final, incorporando tanto a variabilidade dos dados quanto a incerteza acerca dos valores ausentes ^23^. A imputação foi realizada por um processo de equações encadeadas (MICE, do inglês *multivariate imputation by chained equations*), técnica que atua sob a suposição de que, dadas as variáveis usadas no procedimento de imputação, os dados faltantes estão ausentes aleatoriamente (MAR, do inglês *missing at random*), o que significa que a probabilidade de um valor estar faltando depende apenas dos valores observados, e não dos não observados.

Diferentes métodos podem ser utilizados no processo de imputação múltipla. Neste estudo, os valores foram imputados pelo método de correspondência preditiva (PMM, do inglês *predictive mean matching*), que tem como princípio identificar um valor adequado entre os dados completos, mediante critérios de similaridade com a informação faltante ^24^. Para esse propósito, considerando apenas observações com dados completos, foram estimados os parâmetros de um modelo de regressão no qual a variável dependente é aquela com informação faltante. Em seguida, realizou-se seleção aleatória da distribuição preditiva posterior, obtendo-se, assim, novo conjunto de parâmetros a serem utilizados na predição tanto dos valores observados quanto dos faltantes. Para cada caso com valor faltante, selecionam-se k valores observados, cujos valores preditos correspondam àqueles com maior proximidade aos estimados para os dados faltantes. Por fim, o valor a ser imputado é aquele selecionado aleatoriamente entre esses k valores observados. Schenker & Taylor ^24^ não encontraram diferenças significativas em imputações realizadas com valores de k entre 3 e 10, sendo k = 5, o *default* adotado pelos softwares que disponibilizam esse procedimento e, portanto, o valor assumido nessa análise. Importante ressaltar que essa etapa deve ser realizada no processo de geração de cada base de dados completa pela imputação múltipla.

Especificamente, as seguintes etapas foram seguidas:

(1) Por meio da técnica *bootstrap*, 2 mil amostras foram selecionadas do banco de mortalidade do SIM com observações completas e faltantes, levando-se em consideração a estrutura multinível desses dados;

(2) Para cada amostra *bootstrap*, foram gerados cinco bancos de dados completos por meio da imputação múltipla, utilizando as variáveis ano, idade, sexo, raça e UF. O número de imputações igual a cinco é o mais frequentemente utilizado, pois se mostra suficiente para conclusões válidas sem o inconveniente de aumentar em demasia a variância ^25^. Ao final, obtiveram-se 10 mil amostras imputadas do banco de mortalidade por dengue;

(3) Cada banco foi agregado por sexo, idade (15-24 anos, 25-39 anos, 40-49 anos, 50-59 anos, 60- 69 anos, 70-79 anos e ≥ 80 anos), ano, UF e escolaridade (< 8 anos de estudo e ≥ 8 anos de estudo) e compatibilizado com os dados das respectivas populações sob risco estimadas. O ponto de corte da escolaridade (8 anos) foi escolhido por ser a única forma de compatibilizar os bancos de mortalidade e o banco de populações;

(4) Para cada banco agregado, ajustado por um modelo Poisson multinível, os estados federados foram considerados como unidades de segundo nível; e as medidas tomadas dentro dos estados ao longo dos anos como unidades de primeiro nível. Desse modo, considerou-se que as taxas de mortalidade observadas em uma mesma UF são possivelmente correlacionadas. A partir desses modelos, foram estimados taxas, razões de taxas e respectivos IC95% obtidos a partir dos percentis 2,5% e 97,5% da distribuição empírica dessas quantidades.

Para tornar computacionalmente viável a estimativa de todos os parâmetros do modelo em tempo hábil, foi utilizado um modelo Bayesiano, com parâmetros obtidos por aproximação aninhada integrada de Laplace (INLA, do inglês *Integrated Nested Laplace Approximations*) ^26^.

As estimativas das taxas e razões de taxas, considerando ou não a imputação, foram comparadas. Estimaram-se, ainda, as razões de taxas das possíveis interações das variáveis escolaridade e outras. Todos esses resultados são apresentados graficamente na forma de efeitos marginais, ou seja, os efeitos das interações mantendo-se as outras variáveis no “valor médio”.

Todas as análises utilizaram o software R 4.0.4 (http://www.r-project.org), com os pacotes *microdatasus*, *MICE*, *INLA* e *ggplot2*.

### Declaração de ética

O estudo utilizou dados de vigilância em saúde, coletados rotineiramente por meio das DO que alimentam o SIM do Ministério da Saúde. Todas as informações foram analisadas de forma anônima e estão disponíveis de forma aberta para qualquer interessado.

## Resultados

Na população brasileira, a proporção estimada de pessoas com até 8 anos de escolaridade apresentou um declínio no período estudado, passando de 44,2% (n = 64,7 milhões), em 2010, para 34,9%, em 2018 (n = 58,5 milhões).

O número de óbitos por dengue no Brasil entre 2010 e 2018 foi de 4.166 casos, com predominância de indivíduos do sexo masculino (53,3%) e com mais de 80 anos (17%). As taxas de mortalidade foram mais elevadas no Estado de Goiás (6,7 por 1 milhão de habitantes) e Mato Grosso do Sul (5,8 por 1 milhão de habitantes) e menos elevadas em Santa Catarina (sem óbitos) e no Rio Grande do Sul. Na série de anos, o ano de 2015 foi o que apresentou o maior número de óbitos por dengue (21,3%), enquanto 2018 exibiu o menor registro de mortes por dengue (2,5%).

As razões de taxas (RT) entre indivíduos com menor e maior nível de escolaridade no Brasil foram de 2,9 e 3,0 por 1 milhão de habitantes no banco, sem e com imputação, respectivamente. Esses resultados sugerem, em geral, que foi atribuído tempo de escolaridade menor que 8 anos a pessoas com escolaridade não preenchida. Após a imputação dos dados, as RT entre menos e mais escolarizados nas UF deixaram de ser estatisticamente significativas, considerando-se o nível de 5% de significância, com exceção de São Paulo, Rio de Janeiro e Distrito Federal (Tabela 1). Ressalta-se que os IC95% nas UF, com pequeno número de óbitos, foram amplos, apontando para baixa precisão para as estimativas das RT.

**Tabela 1:** Taxa de mortalidade por dengue geral, entre menos e mais escolarizados, com e sem imputação por Unidades Federativas (UF). Brasil, 2010-2018.

UF	Taxa (IC95%)	Sem imputação			Com imputação		
		Taxa (< 8 anos de estudo) (IC95%)	Taxa (≥ 8 anos de estudo) (IC95%)	RT (IC95%)	Taxa (< 8 anos de estudo) (IC95%)	Taxa (≥ 8 anos de estudo) (IC95%)	RT (IC95%)
Rondônia	2,5 (1,7-3,6) *	4,6 (3,1-6,8) *	0,7 (0,3-1,8)	7,0 (0,4-14,4)	7,1 (6,3-26,9) *	1,3 (0,7-3,9)	5,4 (0,5-11)
Acre	1,8 (0,9-3,4)	2,8 (1,3-6,2) *	1,0 (0,3-3,1)	2,8 (1,16,7) *	6,1 (3,7-19,6) *	3,3 (1,7-10) *	1,6 (0,6-3,9)
Amazonas	2,0 (1,5-2,6) *	1,6 (0,9-2,7)	2,2 (1,6-3,1) *	0,7 (0,3-1,2)	2,0 (1,6-6,9) *	2,6 (2,4-9,9) *	0,8 (0,6-0,9)
Roraima	1,0 (0,3-3,1)	2,2 (0,5-8,6)	0,5 (0,1-3,5)	4,4 (0,2-15)	3,2 (2,2-9,7) *	1,0 (0,5-2,9)	2,2 (0,9-6,7)
Pará	1,7 (1,4-2,1) *	2,4 (1,9-3,2) *	1,1 (0,8-1,6)	2,2 (1,2-3,1) *	2,6 (2,5-7,8) *	1,2 (1,1-3,6) *	2,1 (0,6-2,3)
Amapá	2,3 (1,3-4,1) *	3,8 (1,7-8,4) *	1,6 (0,6-3,7)	2,4 (0,5-5,3)	8,8 (5,7-28,2) *	3,4 (1,9-9,9) *	2,2 (0,9-5,1)
Tocantins	2,1 (1,4-3,3) *	3,4 (2,0-5,8) *	1,2 (0,6-2,6)	2,8 (0,3-5,4)	4,7 (3,7-16,9) *	1,9 (1,2-6,3) *	2,2 (0,6-3,7)
Maranhão	1,7 (1,4-2,1) *	2,4 (1,8-3,1) *	1,1 (0,7-1,6)	2,2 (1,1-3,2) *	2,4 (2,4-9,6) *	1,2 (1,1-4,4) *	2,1 (0,4-2,3)
Piauí	1,1 (0,8-1,7)	1,6 (1,0-2,6)	0,6 (0,3-1,3)	2,9 (0,2-5,5)	1,9 (1,7-7,2) *	0,8 (0,6-2,5)	2,5 (0,4-3,3)
Ceará	3,5 (3,0-4,0) *	4,7 (4,0-5,6) *	2,4 (1,9-3,0) *	2,0 (1,4-2,5) *	5,7 (5,4-21,8) *	2,8 (2,6-10,0) *	2,0 (0,6-2,2)
Rio Grande do Norte	5,1 (4,3-6,1) *	7,5 (6,1-9,3) *	2,9 (2,1-4,1) *	2,6 (1,6-3,6) *	9,2 (8,8-27,6) *	3,4 (3,0-10,0) *	2,7 (0,5-3,1)
Paraíba	2,0 (1,5-2,6) *	2,4 (1,7-3,4) *	1,6 (1,0-2,5)	1,5 (0,7-2,4)	3,8 (3,3-13,6) *	2,7 (2,1-8,8) *	1,4 (0,5-1,9)
Pernambuco	3,5 (3,1-4,0) *	5,6 (4,8-6,6) *	1,7 (1,3-2,1) *	3,4 (2,4-4,4) *	6,2 (6,1-24,3) *	1,8 (1,7-6,8) *	3,5 (0,7-3,7)
Alagoas	1,6 (1,1-2,1) *	2,0 (1,3-3,0) *	1,1 (0,6-2,0)	1,8 (0,5-3,2)	3,9 (3,1-11,9) *	2,4 (1,6-7,3) *	1,5 (0,5-2,6)
Sergipe	1,2 (0,8-1,9)	1,5 (0,8-2,7)	0,9 (0,4-1,9)	1,6 (0,1-3,2)	2,0 (1,6-7,1) *	1,3 (0,9-4,2)	1,5 (0,5-2,4)
Bahia	1,2 (1,0-1,4)	1,7 (1,4-2,1) *	0,7 (0,5-1,0)	2,3 (1,4-3,2) *	2,6 (2,4-7,9) *	1,1 (0,9-3,3)	2,3 (0,5-2,9)
Minas Gerais	2,9 (2,7-3,2) *	4,5 (4,0-5,0) *	1,8 (1,5-2,1) *	2,5 (2,0-3,0) *	6,6 (6,3-25,9) *	2,7 (2,4-10,0) *	2,4 (0,8-2,8)
Espírito Santo	3,7 (3,1-4,5) *	5,3 (4,0-6,9) *	2,8 (2,1-3,7) *	1,9 (1,2-2,7) *	8,0 (7,0-30,2) *	4,0 (3,4-15) *	1,9 (0,9-2,4)
Rio de Janeiro	3,1 (2,8-3,5) *	6,5 (5,7-7,3) *	1,7 (1,4-2,0) *	3,9 (3,1-4,7) *	7,3 (7,0-21,9) *	1,9 (1,8-5,6) *	3,9 (1,7-4,1) *
São Paulo	1,9 (1,7-2,0) *	3,9 (3,5-4,3) *	1,0 (0,9-1,1)	4,0 (3,3-4,6) *	5,9 (5,6-17,8) *	1,4 (1,3-4,4) *	4,0 (1,5-4,5) *
Paraná	1,1 (0,9-1,4)	2,2 (1,7-2,8) *	0,5 (0,3-0,7)	4,8 (2,5-7,1) *	2,4 (2,3-9,2) *	0,5 (0,5-1,8)	4,8 (0,9-5,3)
Santa Catarina	-	-	-	-	-	-	-
Rio Grande do Sul	0,0 (0,0-0,1)	0,0 (0,0-0,2)	-	-	0,1 (0,0-0,2)	-	-
Mato Grosso do Sul	5,8 (4,8-7,1) *	8,3 (6,5-11,0) *	4,0 (2,9-5,4) *	2,1 (1,3-2,9) *	8,9 (8,5-26,6) *	4,2 (4,0-13,0) *	2,1 (0,7-2,2)
Mato Grosso	4,9 (4,0-5,9) *	9,3 (7,5-12,0) *	2,1 (1,4-3,0) *	4,5 (2,6-6,5) *	10,1 (9,7-30,6) *	2,4 (2,1-7,1) *	4,3 (0,7-5,0)
Goiás	6,7 (6,0-7,5) *	11,1 (9,7-13,0) *	3,8 (3,1-4,9) *	2,9 (2,2-3,6) *	15,7 (14,8-61) *	5,1 (4,6-19) *	3,0 (0,8-3,5)
Distrito Federal	2,5 (1,9-3,3) *	6,9 (4,9-9,8) *	1,1 (0,7-1,8)	6,2 (2,6-9,9) *	8,2 (7,4-31,2) *	1,4 (1,1-5,0) *	5,7 (2,1-7,0) *
Brasil	2,4 (2,3-2,5) *	3,9 (3,8-4,1) *	1,4 (1,3-1,5) *	2,9 (2,7-3,1) *	5,0 (2,9-7,8) *	1,7 (1,0-2,7)	3,0 (2,7-3,3) *

IC95%: intervalo de 95% de confiança; RT: razão de taxa.

* Taxas e razões de taxa estatisticamente significativas.

As UF com maiores taxas de mortalidade entre os menos escolarizados foram Goiás (15,7 por 1 milhão de habitantes), Mato Grosso (10,1 por 1 milhão de habitantes) e Mato Grosso do Sul (8,9 por 1 milhão de habitantes), ambos localizados na Região Centro-oeste do Brasil.

Para os estados com maior número de óbitos, as RT preditas entre os menos e os mais escolarizados foram semelhantes, comparando-se os dados sem imputação com os dados imputados. Considerando o modelo sem imputação, em alguns estados houve maior discrepância entre a taxa geral de mortalidade e a razão de mortalidade entre os menos e os mais escolarizados, como no Paraná, onde a taxa geral foi baixa (1,1 por 1 milhão de habitantes) e a razão foi maior, e no Mato Grosso do Sul, onde a taxa geral foi alta (5,8 por 1 milhão de habitantes) e a RT foi de 2,1. A maior RT entre menos e mais escolarizados aconteceu no Distrito Federal; e a menor, no Amazonas, levando em consideração o modelo imputado e resultados com significância estatística (Tabela 1).

Analisando os ajustes pelos modelos de Poisson multinível, as estimativas pontuais das RT foram próximas, considerando os dados não imputados e os imputados (Tabela 2). Os intervalos de confiança das RT com base nos dados imputados foram maiores devido ao aumento da incerteza gerado pela inclusão dos valores imputados. Considerando apenas os resultados obtidos para os dados imputados, observamos que as taxas de mortalidade por dengue foram maiores nos anos de 2010 e 2013 e atingiram um pico em 2015, a partir do qual passaram a decrescer. A taxa de mortalidade em homens foi 40% maior que em mulheres. A chance de morrer por dengue aumentou gradativamente com a idade - pessoas com mais de 80 anos apresentaram 14 vezes mais chances de morrer do que jovens de 15-25 anos. Os menos escolarizados tiveram mortalidade 70% maior em comparação aos mais escolarizados.

**Tabela 2:** Modelo estatístico multinível da relação da mortalidade por dengue, considerando ano, sexo, idade e escolaridade. Brasil, 2010-2018.

Variável	Modelo sem imputação	Modelo com imputação
RT (IC95%)	RT (IC95%)
Ano		
2010	1,0	1,0
2011	0,8 (0,7-0,9)	0,7 (0,4-1,3)
2012	0,6 (0,5-0,7)	0,5 (0,3-0,8)
2013	0,9 (0,8-1,0)	0,8 (0,7-1,1)
2014	0,6 (0,5-0,7)	0,6 (0,4-0,8)
2015	1,2 (1,1-1,4)	1,2 (0,6-2,0)
2016	0,8 (0,7-0,9)	0,8 (0,3-1,4)
2017	0,2 (0,1-0,2)	0,1 (0,1-0,2)
2018	0,1 (0,1-0,2)	0,1 (0,1-0,2)
Sexo		
Feminino	1,0	1,0
Masculino	1,3 (1,2-1,4)	1,4 (1,3-1,5)
Idade (anos)		
15-24	1,0	1,0
25-39	1,4 (1,2-1,6)	1,4 (1,2-1,7)
40-49	1,7 (1,4-1,9)	1,7 (1,4-2,1)
50-59	2,4 (2,0-2,7)	2,5 (1,9-3,1)
60-69	2,9 (2,5-3,4)	3,2 (2,5-4,0)
70-79	5,2 (4,5-6,1)	6,0 (4,2-8,2)
≥ 80	11,6 (10,0-13,5)	14,0 (9,0-19,8)
Escolaridade (anos de estudo)		
≥ 8	1,0	1,0
< 8	1,8 (1,6-1,9)	1,7 (1,4-2,1)

IC95%: intervalo de 95% de confiança; RT: razão de taxa.

**Figura 1:**
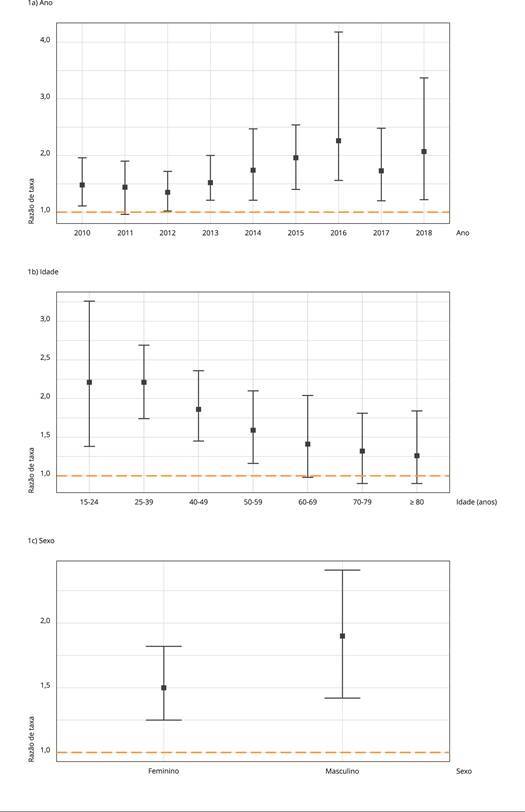
Razões de taxas dos modelos imputados com interação da escolaridade por ano, idade e sexo. Brasil, 2010-2018.

Considerando o modelo de interação do ano com a escolaridade, controlado pelas demais variáveis, as RT entre os menos e os mais escolarizados aumentaram de 2012 a 2016. No modelo de interação da escolaridade com a idade, controlado pelas outras variáveis, o efeito da escolaridade foi maior entre os mais jovens e decaiu com o avançar da idade. No modelo de interação entre sexo e escolaridade, controlado pelas demais variáveis, a RT entre os menos e os mais escolarizados foi maior entre os homens (Figura 1).

## Discussão

Embora a escolaridade geral da população brasileira nos últimos anos tenha aumentado ^27^, observou-se aumento nos diferenciais das taxas de mortalidade por dengue entre os menos e os mais escolarizados no Brasil. As mortes por dengue foram mais frequentes entre homens e suas chances aumentaram com a idade. Entre os mais jovens, identificou-se um diferencial da mortalidade por nível educacional mais pronunciado. São Paulo, Rio de Janeiro e Distrito Federal foram as UF que apresentaram maiores RT entre os menos e os mais escolarizados.

O método de imputação escolhido pouco afetou os coeficientes estimados, mas aumentou a variância das RT. Por essa razão, a maior parte das RT entre os menos e os mais escolarizados deixaram de ser estatisticamente significativas após a imputação dos dados. Apesar disso, o método usado se mostrou vantajoso, pois levou em conta a estrutura hierárquica/multinível dos dados e a incerteza a respeito do não preenchimento da escolaridade. Independentemente do processo de imputação, os resultados mostraram maiores taxas de mortalidade por dengue entre os menos escolarizados, na maioria das UF.

Nas últimas décadas, o nível de escolaridade aumentou principalmente nas regiões mais pobres do país, como Norte e Nordeste ^20^^,^^28^. Esse aumento, contudo, não resultou em redução nos diferenciais de mortalidade por dengue entre os menos e os mais escolarizados. Vários fatores podem ter contribuído para isso. Primeiro, aqueles que mantêm menor nível de escolaridade são os de menor renda, residentes em áreas mais propensas à proliferação do mosquito e, consequentemente, à maior incidência. Nesse contexto, ainda que a letalidade fosse independente de qualquer determinante social, a mortalidade seria maior entre os menos escolarizados. Do ponto de vista individual, um nível maior de educação influenciaria os indivíduos em termos de conhecimento, atitudes e práticas em relação à dengue, de modo que pessoas com maior nível de escolaridade teriam maior capacidade de decisão sobre medidas preventivas, como descarte adequado de recipientes que possam acumular água parada e limpeza regular de áreas propensas à reprodução do mosquito transmissor ^29^. Outro possível fator que se deve levar em consideração diz respeito a repetidas infecções causadas por vírus de sorotipos diferentes, que estão associados à apresentação de dengue hemorrágica e de síndrome de choque da dengue ^30^. Além disso, indivíduos com menor nível de escolaridade tendem a apresentar maior número de condições preexistentes, como diabetes, hipertensão e distúrbios cardiovasculares, aumentando a letalidade da doença ^31^^,^^32^.

O acesso e a utilização dos serviços de saúde também são pontos norteadores do entendimento da mortalidade por dengue e da escolaridade. Há fortes evidências de que o acesso e a utilização desses serviços são bastante desiguais entre os diferentes grupos sociais ^33^^,^^34^^,^^35^. Estudos mostram também que pessoas mais escolarizadas tendem a conseguir mais acesso não somente aos serviços de saúde, mas também aos que apresentam maior qualidade ^36^^,^^37^.

As taxas de mortalidade por dengue foram menores em dois estados da Região Sul, a mais fria do país, fator que dificulta o crescimento e a propagação do vetor da dengue ^38^. No entanto, essa diminuta quantidade de casos nessas UF pode também ser reflexo de subnotificação ^39^^,^^40^. O Distrito Federal, UF com o maior Índice de Desenvolvimento Humano (IDH) do Brasil ^41^, chamou a atenção com uma taxa de mortalidade mediana em comparação com os demais estados, mas com a mais alta RT (embora a precisão tenha sido baixa), ou seja, uma elevada mortalidade dos menos escolarizados, o que pode indicar significativa desigualdade no acesso aos serviços de saúde. No entanto, as diferenças por estado não guardaram relação aparente com o índice de Gini aferido em 2010 ^42^, embora esse indicador não aborde o setor saúde e o acesso a ele.

A maioria dos estudos aponta o sexo feminino como predominante em número de casos e mortes por dengue ^43^. Outras pesquisas afirmam que há maior predomínio de mulheres no número de casos, mas que a maioria das mortes por dengue ocorre com homens ^44^. Os resultados encontrados neste estudo indicam o predomínio da mortalidade no sexo masculino, mesmo com a escolaridade sendo considerada na análise. Pesquisas apontam que mulheres são mais propensas a buscar serviços de saúde ^45^.

O aumento do efeito da escolaridade na interação com o ano entre os anos de 2012 e 2016 pode resultar do aumento no número de casos nesse período, principalmente em 2015 e 2016, quando houve a introdução de outras arboviroses transmitidas pelo mesmo vetor no cenário nacional, que em aspectos clínicos, se assemelham com a dengue e acometem de forma semelhante os mesmos grupos sociais, Zika e chikungunya ^46^^,^^47^. O aumento da incidência foi mais pronunciado nas populações mais vulneráveis, ocasionando um aumento nos diferenciais de mortalidade entre os menos e os mais escolarizados. Outro ponto a ser considerado diz respeito à precocidade do entendimento desses eventos à época, uma vez que esses agravos podem ter sido registrados no SIM erroneamente como dengue ^43^.

A taxa de mortalidade por dengue apresentou uma relação direta com a idade, em consonância com achados de outros estudos ^6^^,^^48^. Porém, os diferenciais de mortalidade diminuíram à medida que a idade avançava, não sendo estatisticamente significativos entre idosos. Isso pode ter decorrido do fato de os idosos apresentarem maior risco de múltiplas morbidades e, assim, ao interagirem com a dengue, estão mais suscetíveis à ocorrência de óbito, ou seja, o efeito protetor da escolaridade nesse segmento populacional é reduzido ^49^.

As principais limitações deste estudo devem-se ao fato de estarmos trabalhando com dados obtidos de forma secundária. Possíveis óbitos por dengue podem não ter sido notificados no sistema devido à falta de vigilância oportuna em saúde ou à falta de acesso, variando de local para local do país. Além disso, a incompatibilidade das informações dos bancos de dados do sistema de mortalidade e dos bancos populacionais limitou o uso de maior número de categorias para descrever a escolaridade. Possivelmente, o impacto da educação seria maior se pudéssemos considerar os que têm curso superior, pois no Brasil há forte relação entre curso superior e melhores condições de vida ^18^.

Os pontos fortes do estudo foram a utilização de base populacional, apesar das limitações descritas anteriormente, a especificação correta do modelo estatístico usado, levando-se em conta a correlação intrínseca dos dados, e a possível aplicabilidade da mesma metodologia para outras doenças, desde que não sejam restritas a populações específicas.
